# 0863. Lithium pharmacokinetics in the rat according to the three different modalities of human poisoning

**DOI:** 10.1186/2197-425X-2-S1-P65

**Published:** 2014-09-26

**Authors:** A-S Hanak, L Chevillard, S El-Bakhli, P Risède, K Peoc'h, B Megarbane

**Affiliations:** INSERM U 1144, Paris-Descartes University, Paris, France; Lariboisière Hospital, INSERM U 1144, Paris-Diderot University, Paris, France

## Introduction

Lithium-related neurological toxicity may be severe resulting in seizures, myoclonic encephalopathy, and coma. Three different poisoning presentations exist in humans, including acute poisoning in non-previously treated patients (A), acute-on-chronic poisoning (A/C), and therapeutic overdose (T). The exact reasons why severity and features are different between these three presentations are unknown, although differences in brain lithium distribution have been suggested.

## Objectives

Our objective was to study lithium pharmacokinetics in blood and brain in rat models corresponding to each human presentation.

## Methods

Development of three models of lithium intoxication in Sprague Dawley rats: A (one intraperitoneal injection of 185 mg/kg Li_2_CO_3_); A/C (800 or 1600 mg/L Li_2_CO_3_ in the drinking water followed by one intraperitoneal injection of 185 mg/kg Li_2_CO_3_ at day 28); T (K_2_Cr_2_O_7_-induced acute renal failure on day 1 followed by intraperitoneal injections of 74 mg/kg/day Li_2_CO_3_ during 5 days); determination of plasma, erythrocyte, cerebrospinal fluid, and brain lithium concentrations using inductively coupled plasma atomic emission spectroscopy (quantification threshold: 0.6 nmol/L); modeling and determination of pharmacokinetics parameters; comparisons with non-parametric tests.

## Results

Lithium followed a tricompartmental pharmacokinetics with a shortened plasma half-life in case of previous chronic exposure (1.73 vs. 3.85h). The peak lithium concentration was measured at 6h in erythrocytes, 2h in cerebrospinal fluid, and 24h in the brain in both A and A/C models; however, the elimination constants k21 (erythrocytes-to-plasma) and k31 (brain-to-plasma) were lower in the A/C model (0.36 *versus* 0.56 and 2.1 *versus* 9.2, respectively), suggesting lithium accumulation. The brain distribution was not homogeneous, with rapid entrance (as soon as 15min), peak at 24h, and delayed elimination (>78h). Lithium accumulation into the brain was more marked in the presence of previous chronic exposure (brain-to-plasma ratio at 54h: 131.27 vs. 6.42; p< 0.0001). Similarly, alteration in renal elimination resulted in increased brain distribution (brain-to-plasma ratio: 10.98 vs. 6.88).

## Conclusions

Our experimental models suggest that the three different presentations of lithium poisonings in humans differ due to lithium blood pharmacokinetics and brain distribution. However, the hypothesis of an additional variability related to different interactions of lithium with neurological targets in each presentation could not be ruled out.

